# Unraveling bovin phylogeny: accomplishments and challenges

**DOI:** 10.1186/1741-7007-8-50

**Published:** 2010-04-29

**Authors:** Faysal Bibi, Elisabeth S Vrba

**Affiliations:** 1 Institut de Paléoprimatologie et Paléontologie humaine: Evolution et Paléoenvironnements CNRS UMR 6046, Université de Poitiers, 40 av. du Recteur Pineau, 86022, Poitiers, France; 2 Department of Geology & Geophysics, PO Box 208109, Yale University, Connecticut 06520, USA

## Abstract

The phylogenetic systematics of bovin species forms a common basis for studies at multiple scales, from the level of domestication in populations to major cladogenesis. The main big-picture accomplishments of this productive field, including two recent works, one in *BMC Genomics*, are reviewed with an eye for some of the limitations and challenges impeding progress. See Research article http://www.biomedcentral.com/1471-2164/10/177

## 

Recent years have seen a flurry of interest and inquiry into the evolutionary history of Bovini, the clade comprising living oxen (genus *Bos*) and the living buffaloes (genera *Syncerus *and *Bubalus*). A disproportionate interest in the phylogenetics of Bovini, relative to other bovid or ruminant clades, no doubt stems from the importance of bovins as a prime source of human sustenance since at least the Pleistocene. In addition, bovins are widespread (naturally occurring on four continents), ecologically differentiated with wide habitat tolerances (Figure 1), taxonomically diverse (around a dozen living species and over 50 fossil), and, given their large size and affinity for wet habitats, possess a high potential for preservation in the fossil record. For these reasons, the evolutionary record of Bovini provides an exemplary resource for studies on evolutionary patterns and processes.

**Figure 1:**
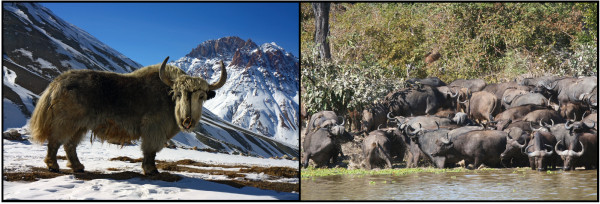
**Bovins stand apart from other antelopes (Bovidae) in the wide range of environments they inhabit, from high montane to wet tropical**. Bovini today comprises 12 species found on four continents. (Yak: iStockphoto.com/kodda; African buffaloes: iStockphoto.com/dawnn).

One avenue of research that has been continuously providing new information, from Miocene higher clade origins to Holocene population dynamics, has been the analysis of bovin genomes. Deeper and more extensive mining of the genomes of bovin species has seen a consistently improving phylogeny for this clade. A recent study by MacEachern *et al*. [1] used 84 autosomal gene sequences from 15 different genes to examine phylogenetic relationships among bovin species and populations, reconstructing geographic divergences and intricate histories of genetic introgression and geographic divergence. Similarly, Decker *et al*. [2] used a much expanded genomic data set to investigate phylogenetic relationships from the level of Ruminantia (ruminants, including cattle, antelope, deer, giraffe, and chevrotains) to that of domesticated cattle breeds, including DNA from the extinct *Bison priscus*. Advances in DNA sequencing techniques have even produced a complete mitochondrial genome from the aurochs (*Bos primigenius*) [3], the progenitor of domestic cattle (*Bos taurus*).

Studies such as these highlight the current focus on unraveling the history of bovin evolution by way of the bovin genome, and the present interest in tracing the history and geography of domestication events. However, two aspects that have seen little progress in the last few years are the phylogenetic assembly of the total bovin clade and the dating of major cladogenetic events within Bovini. Even in the cutting edge studies cited above, little advance is made at the level of major bovin cladogenesis over previous work almost a decade older. From this perspective, the literature of the last years has provided mostly incomplete phylogenies of Bovini dated with inadequate molecular clock estimates. This stems from several factors. First has been the dearth of phylogenetic work on fossil bovin taxa. Second is the regular omission of certain crucial bovin taxa from phylogenetic work. Third is a lack of precision in the phylogenetic terminology used to communicate between paleontological and molecular studies, resulting in the choice of poor references for molecular clock calibration.

We here present our views to highlight some significant gaps and challenges that remain in the field of bovin phylogenetics. Our recommendations also aim to increase the utility of studies for workers of different methodological backgrounds.

## Stability in bovin systematics: mission accomplished?

**Figure 2:**
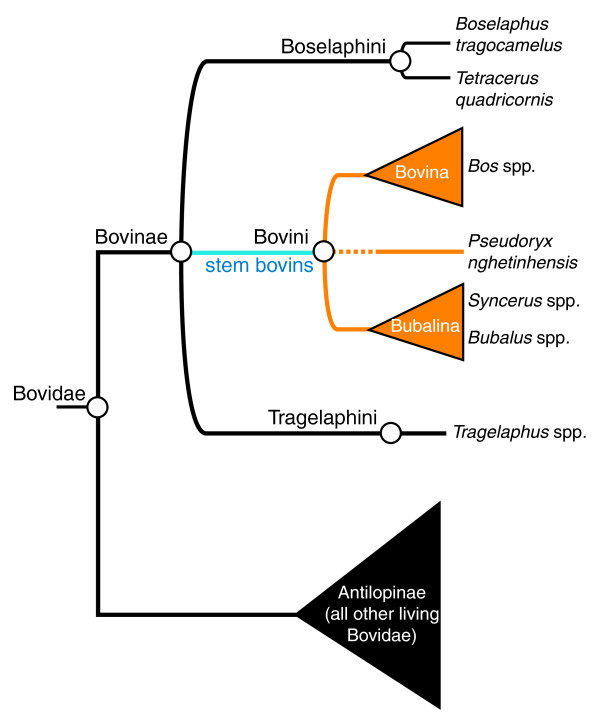
**Phylogeny showing the position of Bovini in Bovidae and Bovinae, the division of Bovini into Bovina and Bubalina, and the uncertain placement of the saola (*Pseudoryx nghetinhensis*)**.

A key achievement of the past two decades of molecular phylogenetic analyses has been recurrent and consistent support for a systematic classification of Bovidae (Figure 2; and see [4]). All living bovids may be divided among either Bovinae or Antilopinae. Bovinae is the clade uniting Bovini (buffaloes and oxen), Tragelaphini (spiral-horned antelopes, including kudu), and Boselaphini (nilgai and chousinga). This classification requires that members of Bovinae be referred to as bovines, while members of Bovini are bovins, though most of the literature still uses the term bovine in reference to the Bovini. Living bovins are further divided among the Bovina (genus *Bos*, including *Bison*) and the Bubalina (*Syncerus *and *Bubalus*). A certain amount of confusion surrounding the taxonomy of domestic derivates of wild bovin species has also been addressed by a ruling by the International Commission on Zoological Nomenclature (see [5]).

One major remaining conundrum concerns the saola (*Pseudoryx nghetinhensis*), a little-understood forest antelope from Vietnam and Laos only made known to science since 1993. This species is challenging the definition, composition, and diagnosis of parts of the bovin phylogenetic tree. The saola's small size, simple non-divergent horns, large preorbital fossa (bony depression anterior to the orbital cavity), and simple-shaped teeth make it a very primitive-looking bovid, especially with respect to the large and derived Bovini. It is therefore puzzling that molecular phylogenetic analyses consistently place the saola within Bovini [6,7]. The exact position and relationship of the saola to the other bovin species is in need of further confirmatory work. Knowing the relationship of this goat-like ungulate to the remainder of Bovini is important, not just to appease curiosity about an enigmatic forest antelope, but because the phylogenetic position of this creature may in fact upset some of the 'stabilized' topology shown in Figure 2 (see also below).

## Dating phylogenies: putting the cow before the cart

Phylogenetic studies of fossil Bovini are a crucially missing basis for bovin molecular clock calibrations. Molecular phylogenies are being calibrated using fossil data that are wrong or, at best, highly speculative. For example, reference calibration nodes used in the literature include: the first appearance of Bovidae, the divergence of *Bison *and *Bos*, the divergence of Bovina from Bubalina, and the divergence of Bovini from Tragelaphini. However, the reality is that none of the ages of these divergence events is at all well established (Geraads's 1992 work [8] might be the only extensive phylogenetic analysis of fossil and living bovins to date). The phylogenetic relationships of many fossil Bovini, and, as a result, the evolutionary history of many living Bovini, await more thorough analysis of fossil bovin taxa.

**Figure 3:**
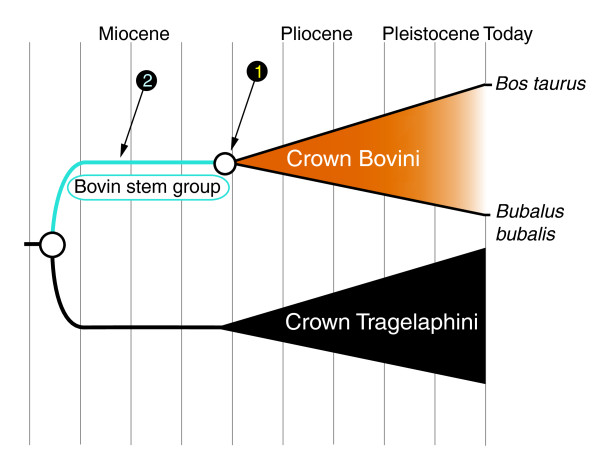
**Fossil taxa relied upon for molecular clock calibrations may produce highly spurious dates if their phylogenetic position is not precisely known**. In this example, an early fossil taxon attributed simply to 'Bovini' might be assumed to be close to the most recent common ancestor of the living bovins (point 1, node of origin of crown Bovini as defined by the most recent common ancestor of *Bos taurus *and *Bubalus bubalis*) when in fact it may be a stem bovin of much older age (point 2).

A related issue concerning the use of fossils to calibrate molecular phylogenies stems from an inattention to the distinction between dating crown clades and total clades [9]. A crown clade is one defined on the basis of extant taxa, while a total clade includes the crown plus any extinct taxa located on the ancestral 'stem' of the crown clade (Figure 3). Despite much progress towards stability in clade names, there is still a certain fog of confusion, much of it gone unnoticed, about clade definitions and compositions. For example, a late Miocene fossil is assigned to 'Bovini' in the paleontological literature without determining whether it actually belongs to the stem group or the crown clade. In a later study, this same taxon is then simply assumed to belong in the crown clade, and is used to date the node of origination of crown Bovini in a molecular phylogeny. The fact that the referenced fossil taxon is just as likely to be a stem bovin means the clade origination estimate might actually be off by millions of years, a wide margin when considering Neogene taxa (Figure 3). The use of poorly-placed fossils to date a molecular phylogeny can only be expected to produce spurious results. Studies seeking to assess the timing of evolutionary events with respect to environmental changes, for example, cannot rely on such results, nor could phylogeographic reconstructions.

Until better paleontological studies are available, caution and wide margins of error are advised when referring to the ages and phylogenetic placement of fossil bovins for molecular clock calibration. Though commendable efforts have been made in this regard, dates produced from molecular clock estimates will remain very imprecise so long as the fossils used to calibrate these rates are themselves poorly understood phylogenetically. The fossil record of Bovini is already very large and holds great promise for dating the major cladogenetic events within Bovini, so there is every reason for rapid progress in this regard.

## From genes to genera: reconciling scales of analysis

The phylogeny of Bovini is being approached from the scale of genes to genera. Studies of the genomes and studies of the fossil record proceed from different methodologies and at different scales, fossils providing a relatively coarse but deep temporal perspective, and molecular work providing a narrow but very highly resolved picture of the modern. Both fields share a common goal, but too often the results of each approach are difficult to reconcile with the other. One limitation to the synthesis of results is the use of limited taxonomic sampling in a phylogenetic analysis. Limited taxonomic representation decreases the reliability and precision of a phylogeny, in turn limiting interpretations on dating, biogeography, and cladogenesis.

Paleontological studies should consider living taxa to the greatest extent they can. This is important considering that workers seeking to calibrate molecular phylogenies are ploughing the literature for information on the timing of origination of crown (that is, extant) clades. Likewise, phylogenetic studies treating living taxa are most informative when they too consider the largest available sample of species. For example, paleontologists investigating the split of Bovina and Bubalina will want to refer to phylogenies that include the entirety of living Bovini to be sure that the node defining the crown clade has been defined to the finest degree possible. Additionally, taxa that are rare or poorly understood have the greatest potential to disrupt 'stable' topologies and alter previous notions, and yet such 'enigmatic' taxa are regularly missing from analyses. An investigation seeking to unravel the relationships among the different clades of Ruminantia cannot afford to omit the Moschidae (musk deer, small ruminants lacking antlers and today restricted to central and northeastern Asia). Any analysis of the Bovini cannot now afford to exclude the saola. It is precisely the fact that such taxa are so disparate in form and restricted in distribution that makes them most interesting for evolutionary reconstruction.

Great progress has been made towards stable systematic classifications in recent years by workers in different fields utilizing different approaches. Continued progress requires an effort to better integrate the different results of the geneticists, ecologists, archaeologists, and paleontologists working on the origins of bovins. Improved communication among workers in different fields will greatly promote the output, precision, and accuracy of results in studies to come.
